# Supplementary material for: A Likelihood Approach for Real-Time Calibration of Stochastic Compartmental Epidemic Models
Source: PLoS Comput Biol. 2017 Jan 17;13(1):e1005257. doi: 10.1371/journal.pcbi.1005257 (PMC5240920; doi:10.1371/journal.pcbi.1005257)
Supplement: S1 File — (TAR.GZ) [file pcbi.1005257.s014.tar.gz › HSPH_Online-SI-Revision/Additions/MSS11/n100-extreme-SEITR_noisy/n100-extreme-SEITR_noisy_NEW_table8.pdf]

1	R0	{1.38654, 9.85565, 0.1}	{2.48143, 15.7026, 0.}	{{2.48143, 15.7026, 0.}, {2.48143, 15.7026, 0.}}	{78.9653, 59.3258, 100.}	inf	658.	2937.32	{2886.56, 2988.23}	346.401	0.64969	newly	368.386	1123.78	{1089., 1156.}	205.055	0.484246	Reff	0.108483	0.301987	{0.301234, 0.302634}	178.373	ar	92379.	89016.7	{88941., 89083.}	3.63964
2	R0	{2.36629, 12.2029, 0.1}	{2.70795, 10.355, 0.}	{{2.70795, 10.355, 0.}, {2.70795, 10.355, 0.}}	{14.4388, 15.1435, 100.}	inf	689.	697.937	{673.037, 728.702}	2.56656	0.0110043	newly	300.	364.89	{336., 388.}	21.63	0.0844184	Reff	0.0235682	0.231988	{0.231571, 0.232428}	884.323	ar	99022.	91636.9	{91604., 91667.}	7.45805
3	R0	{2.32253, 12.836, 1.}	{2.91946, 11.8546, 2.}	{{2.91946, 11.8546, 2.}, {2.91946, 11.8546, 2.}}	{25.7015, 7.64553, 100.}	inf	1063.	1246.99	{1205.12, 1281.41}	17.3086	0.0692168	newly	463.	584.	{557., 612.}	26.1339	0.100549	Reff	0.0245491	0.199461	{0.198977, 0.199954}	712.498	ar	98997.	93468.9	{93435., 93504.}	5.58416
4	R0	{1.79075, 11.2058, 1.}	{2.74056, 10.6768, 0.}	{{2.74056, 10.6768, 0.}, {2.74056, 10.6768, 0.}}	{53.0397, 4.72117, 100.}	inf	828.	834.306	{803.304, 858.516}	2.21446	0.00955355	newly	412.	428.93	{402., 456.}	5.23058	0.0219855	Reff	0.0574114	0.227145	{0.226669, 0.227575}	295.644	ar	96920.	91951.4	{91919., 91991.}	5.12653
5	R0	{2.26952, 9.62371, 0.1}	{2.77394, 8.24535, 0.}	{{2.77394, 8.24535, 0.}, {2.77394, 8.24535, 0.}}	{22.2258, 14.3225, 100.}	inf	187.	158.247	{145.263, 171.562}	15.3757	0.0734788	newly	78.	98.5	{85., 110.}	26.2821	0.0994554	Reff	0.0257137	0.215785	{0.215582, 0.215983}	739.186	ar	98881.	92266.	{92251., 92284.}	6.68988
6	R0	{1.57191, 6.89004, 0.1}	{2.99092, 10.3743, 0.}	{{2.99092, 10.3743, 0.}, {2.99092, 10.3743, 0.}}	{90.2727, 50.5697, 100.}	inf	54.	479.026	{455.085, 501.341}	787.085	0.947637	newly	40.	246.15	{226., 267.}	515.375	0.788282	Reff	0.077951	0.183091	{0.182653, 0.183414}	134.88	ar	95046.	93984.7	{93962., 94011.}	1.11661
7	R0	{1.99226, 17.2339, 1.}	{2.88523, 18.2127, 0.}	{{2.88523, 18.2127, 0.}, {2.88523, 18.2127, 0.}}	{44.8219, 5.67928, 100.}	inf	2865.	3626.21	{3572.86, 3683.48}	26.5692	0.102298	newly	982.	1199.66	{1151., 1239.}	22.165	0.0867617	Reff	0.0456427	0.223685	{0.222931, 0.224313}	390.078	ar	97992.	93205.4	{93143., 93253.}	4.88465
8	R0	{2.63369, 15.1879, 0.1}	{2.80387, 10.6536, 0.}	{{2.80387, 10.6536, 0.}, {2.80387, 10.6536, 0.}}	{6.46153, 29.8546, 100.}	inf	1428.	659.224	{627.075, 687.818}	53.8358	0.33594	newly	510.	337.3	{311., 361.}	33.8627	0.180234	Reff	0.0154861	0.214346	{0.213913, 0.214785}	1284.12	ar	99451.	92527.5	{92493., 92561.}	6.96175
9	R0	{2.53271, 9.90552, 0.1}	{2.77394, 8.24535, 0.}	{{2.77394, 8.24535, 0.}, {2.77394, 8.24535, 0.}}	{9.52454, 16.76, 100.}	inf	171.	156.987	{143.727, 167.98}	8.5047	0.0392689	newly	96.	97.52	{87., 109.}	7.04167	0.0302317	Reff	0.0167159	0.215806	{0.215559, 0.216083}	1191.02	ar	99355.	92262.1	{92247., 92280.}	7.13895
10	R0	{2.48791, 18.9003, 2.}	{2.86001, 13.5542, 0.}	{{2.86001, 13.5542, 0.}, {2.86001, 13.5542, 0.}}	{14.9562, 28.2856, 100.}	inf	3761.	2008.54	{1970.65, 2044.89}	46.5957	0.272475	newly	1167.	853.75	{824., 883.}	26.8423	0.135926	Reff	0.0212716	0.215015	{0.214119, 0.215692}	910.805	ar	99291.	93006.6	{92955., 93050.}	6.32922
11	R0	{2.84414, 18.4765, 1.}	{2.52588, 12.2207, 0.}	{{2.52588, 12.2207, 0.}, {2.52588, 12.2207, 0.}}	{11.1899, 33.858, 100.}	inf	3141.	1662.95	{1624.02, 1704.21}	47.0567	0.276267	newly	1003.	780.35	{746., 812.}	22.1984	0.109239	Reff	0.0117463	0.278281	{0.277666, 0.278918}	2269.1	ar	99654.	89599.8	{89545., 89656.}	10.0891
12	R0	{2.29369, 14.622, 0.1}	{2.73634, 13.3345, 1.}	{{2.73634, 13.3345, 1.}, {2.73634, 13.3345, 1.}}	{19.2987, 8.80542, 900.}	inf	1365.	1704.59	{1675.45, 1734.77}	24.8782	0.0964389	newly	542.	735.93	{707., 763.}	35.7804	0.132615	Reff	0.0247948	0.235004	{0.234467, 0.235486}	847.796	ar	98982.	91920.9	{91881., 91976.}	7.13369
13	R0	{2.73413, 16.7529, 1.}	{2.9884, 12.8326, 0.}	{{2.9884, 12.8326, 0.}, {2.9884, 12.8326, 0.}}	{9.29971, 23.4007, 100.}	inf	2050.	1318.38	{1285.15, 1344.76}	35.6889	0.191787	newly	706.	578.74	{549., 604.}	18.0255	0.0865712	Reff	0.0138347	0.189225	{0.188666, 0.189794}	1267.75	ar	99543.	93966.8	{93932., 94004.}	5.60176
14	R0	{1.79554, 16.5943, 2.}	{2.56757, 17.005, 0.}	{{2.56757, 17.005, 0.}, {2.56757, 17.005, 0.}}	{42.9973, 2.47477, 100.}	inf	3777.	4127.	{4071.89, 4183.71}	9.26653	0.0384635	newly	1313.	1472.3	{1427., 1511.}	12.1325	0.0496147	Reff	0.0662734	0.293762	{0.292882, 0.294619}	343.258	ar	96821.	90107.5	{90026., 90184.}	6.93395
15	R0	{1.91429, 13.7771, 1.}	{2.88523, 18.2127, 0.}	{{2.88523, 18.2127, 0.}, {2.88523, 18.2127, 0.}}	{50.7205, 32.1952, 100.}	inf	1469.	3625.6	{3567.56, 3670.85}	146.808	0.392334	newly	621.	1206.1	{1170., 1250.}	94.219	0.28814	Reff	0.0501544	0.223719	{0.222946, 0.224498}	346.06	ar	97541.	93200.5	{93139., 93257.}	4.44988
16	R0	{1.42094, 10.5871, 2.}	{2.93371, 17.1903, 1.}	{{2.93371, 17.1903, 1.}, {2.93371, 17.1903, 1.}}	{106.462, 62.3702, 50.}	inf	1201.	3783.11	{3733.75, 3837.77}	214.996	0.49828	newly	644.	1315.14	{1271., 1359.}	104.214	0.309941	Reff	0.103388	0.216582	{0.215869, 0.217332}	109.485	ar	93044.	93572.9	{93503., 93634.}	0.568484
17	R0	{1.45515, 6.16466, 0.1}	{2.17292, 6.16453, 0.}	{{2.17292, 6.16453, 0.}, {2.17292, 6.16453, 0.}}	{49.3258, 0.0020887, 100.}	inf	50.	106.037	{93.1345, 119.884}	112.075	0.324629	newly	41.	85.67	{72., 100.}	108.951	0.317035	Reff	0.0982954	0.352893	{0.352589, 0.353174}	259.013	ar	93254.	83815.9	{83795., 83838.}	10.1209
18	R0	{2.85669, 13.2416, 0.1}	{2.9194, 10.1535, 0.}	{{2.9194, 10.1535, 0.}, {2.9194, 10.1535, 0.}}	{2.19533, 23.3211, 100.}	inf	778.	483.225	{462.521, 501.662}	37.8888	0.207068	newly	314.	255.43	{236., 277.}	18.6529	0.090372	Reff	0.0106269	0.193973	{0.193696, 0.194232}	1725.31	ar	99644.	93469.6	{93445., 93493.}	6.19644
19	R0	{2.56417, 17.9558, 0.1}	{2.7499, 15.3124, 0.}	{{2.7499, 15.3124, 0.}, {2.7499, 15.3124, 0.}}	{7.24317, 14.7216, 100.}	inf	3203.	3573.	{3516.51, 3629.81}	11.5516	0.0474499	newly	1136.	1379.22	{1334., 1423.}	21.4102	0.0841246	Reff	0.01818	0.248805	{0.24805, 0.249564}	1268.57	ar	99389.	92032.9	{91963., 92094.}	7.40135
20	R0	{2.27517, 11.1864, 0.1}	{2.99092, 10.3743, 0.}	{{2.99092, 10.3743, 0.}, {2.99092, 10.3743, 0.}}	{31.459, 7.25957, 100.}	inf	460.	480.135	{456.695, 500.652}	4.83023	0.0203222	newly	219.	248.69	{231., 265.}	13.5936	0.0546798	Reff	0.0255502	0.183018	{0.182663, 0.183393}	616.309	ar	98894.	93987.4	{93967., 94009.}	4.96152
21	R0	{2.35735, 10.4735, 0.1}	{2.77394, 8.24535, 0.}	{{2.77394, 8.24535, 0.}, {2.77394, 8.24535, 0.}}	{17.6719, 21.2741, 100.}	inf	299.	159.448	{143.227, 173.164}	46.6729	0.274103	newly	156.	100.22	{86., 112.}	35.7564	0.194254	Reff	0.0230549	0.21577	{0.215534, 0.21597}	835.897	ar	99036.	92267.2	{92252., 92281.}	6.83472
22	R0	{1.79551, 11.9179, 2.}	{2.73634, 13.3345, 1.}	{{2.73634, 13.3345, 1.}, {2.73634, 13.3345, 1.}}	{52.3991, 11.8861, 50.}	inf	886.	1702.64	{1667.65, 1739.55}	92.1715	0.283626	newly	400.	733.69	{707., 765.}	83.4225	0.263227	Reff	0.0555172	0.234977	{0.234293, 0.235571}	323.251	ar	97033.	91912.5	{91863., 91958.}	5.27708
23	R0	{1.23545, 6.31687, 0.1}	{2.26582, 8.12562, 0.}	{{2.26582, 8.12562, 0.}, {2.26582, 8.12562, 0.}}	{83.4007, 28.6336, 100.}	inf	99.	232.957	{216.035, 250.778}	135.31	0.370905	newly	79.	151.54	{134., 167.}	91.8228	0.281487	Reff	0.139655	0.328054	{0.327745, 0.328387}	134.903	ar	88739.	85635.2	{85602., 85665.}	3.49763
24	R0	{2.24089, 10.1789, 0.1}	{2.93906, 10.3908, 0.}	{{2.93906, 10.3908, 0.}, {2.93906, 10.3908, 0.}}	{31.1558, 2.08158, 100.}	inf	347.	569.323	{540.56, 594.921}	64.0701	0.214733	newly	171.	294.64	{272., 316.}	72.3041	0.235597	Reff	0.025703	0.191523	{0.191098, 0.191927}	645.138	ar	98868.	93619.4	{93596., 93643.}	5.30868
25	R0	{1.57298, 10.2607, 0.1}	{2.52588, 12.2207, 0.}	{{2.52588, 12.2207, 0.}, {2.52588, 12.2207, 0.}}	{60.5796, 19.1023, 100.}	inf	870.	1656.61	{1616.61, 1700.69}	90.4144	0.279615	newly	443.	776.07	{742., 808.}	75.1851	0.243266	Reff	0.0819837	0.278265	{0.27761, 0.278944}	239.415	ar	94957.	89596.7	{89541., 89650.}	5.64498
26	R0	{1.2154, 8.2576, 0.1}	{2.51572, 13.5261, 0.}	{{2.27199, 11.6669, 0.}, {2.7499, 15.3124, 0.}}	{106.987, 63.8023, 100.}	inf	525.	2013.76	{1448.98, 2555.5}	283.573	0.568178	newly	345.	856.25	{711., 1003.}	148.188	0.390091	Reff	0.143952	0.289001	{0.2389, 0.34099}	100.762	ar	88421.	88957.7	{85711., 92076.}	3.57466
27	R0	{2.22841, 18.7983, 2.}	{2.7499, 15.3124, 0.}	{{2.7499, 15.3124, 0.}, {2.7499, 15.3124, 0.}}	{23.4018, 18.5436, 100.}	inf	3488.	2532.75	{2486.42, 2571.4}	27.3868	0.139022	newly	1079.	976.85	{937., 1014.}	9.4671	0.0433797	Reff	0.0322005	0.239458	{0.238836, 0.240098}	643.647	ar	98756.	92043.5	{91993., 92094.}	6.79707
28	R0	{1.27778, 5.36767, 1.}	{2.68628, 9.02342, 1.}	{{2.68628, 9.02342, 1.}, {2.68628, 9.02342, 1.}}	{110.23, 68.1068, 0.}	inf	34.	316.407	{297.008, 337.259}	830.61	0.968154	newly	32.	183.4	{168., 197.}	473.125	0.757144	Reff	0.130065	0.233088	{0.232765, 0.23343}	79.2086	ar	89843.	91417.7	{91395., 91442.}	1.75271
29	R0	{2.38042, 15.8901, 2.}	{2.71225, 12.7047, 1.}	{{2.71225, 12.7047, 1.}, {2.71225, 12.7047, 1.}}	{13.9399, 20.0464, 50.}	inf	2325.	1816.3	{1777.1, 1856.52}	21.8796	0.107291	newly	849.	820.64	{788., 855.}	3.93875	0.0175516	Reff	0.0237804	0.240455	{0.239796, 0.241109}	911.146	ar	99112.	91690.6	{91644., 91737.}	7.48785
30	R0	{2.53188, 12.7607, 0.1}	{2.99092, 10.3743, 0.}	{{2.99092, 10.3743, 0.}, {2.99092, 10.3743, 0.}}	{18.1302, 18.701, 100.}	inf	1003.	831.009	{801.5, 858.217}	17.1477	0.0818412	newly	428.	431.72	{409., 455.}	3.61215	0.015589	Reff	0.0182295	0.185439	{0.184946, 0.185945}	917.245	ar	99317.	93986.2	{93957., 94014.}	5.36746
31	R0	{1.24482, 10.5471, 1.}	{2.44868, 15.3948, 0.}	{{2.30907, 14.0824, 0.}, {2.48143, 15.7026, 0.}}	{96.7094, 45.962, 100.}	inf	1280.	2831.69	{2368.06, 2984.07}	121.226	0.34328	newly	689.	1101.61	{1002., 1159.}	59.8853	0.203207	Reff	0.142009	0.309198	{0.301284, 0.340674}	117.731	ar	89097.	88521.8	{86406., 89076.}	0.649169
32	R0	{2.30446, 17.9607, 1.}	{2.86118, 15.5722, 2.}	{{2.86118, 15.5722, 2.}, {2.86118, 15.5722, 2.}}	{24.1586, 13.2987, 100.}	inf	3363.	2955.78	{2907.56, 2997.08}	12.109	0.0560856	newly	1094.	1119.99	{1084., 1154.}	2.80165	0.011926	Reff	0.0266626	0.222493	{0.221769, 0.223186}	734.476	ar	99028.	93018.6	{92958., 93079.}	6.06837
33	R0	{2.31579, 11.8236, 2.}	{2.77394, 8.24535, 0.}	{{2.77394, 8.24535, 0.}, {2.77394, 8.24535, 0.}}	{19.7837, 30.2636, 100.}	inf	477.	158.989	{143.894, 170.281}	66.669	0.478023	newly	197.	98.98	{88., 110.}	49.7563	0.300525	Reff	0.0234821	0.215744	{0.215501, 0.215965}	818.758	ar	99012.	92267.7	{92254., 92283.}	6.81156
34	R0	{1.6678, 10.6744, 1.}	{2.71225, 12.7047, 1.}	{{2.71225, 12.7047, 1.}, {2.71225, 12.7047, 1.}}	{62.6243, 19.0204, 0.}	inf	572.	1194.14	{1157.25, 1225.49}	108.765	0.319564	newly	278.	535.19	{513., 563.}	92.5144	0.284204	Reff	0.0712985	0.235279	{0.234709, 0.235836}	229.991	ar	95809.	91680.9	{91638., 91714.}	4.30873
35	R0	{1.65476, 7.07559, 0.1}	{2.77394, 8.24535, 0.}	{{2.77394, 8.24535, 0.}, {2.77394, 8.24535, 0.}}	{67.6339, 16.5324, 100.}	inf	69.	156.845	{139.549, 170.11}	127.311	0.355631	newly	50.	97.84	{88., 110.}	95.68	0.289767	Reff	0.0705259	0.215753	{0.21552, 0.215981}	205.92	ar	95746.	92265.7	{92251., 92282.}	3.63489
36	R0	{2.71807, 18.5959, 0.1}	{2.53075, 11.6597, 0.}	{{2.53075, 11.6597, 0.}, {2.53075, 11.6597, 0.}}	{6.89168, 37.2995, 100.}	inf	3776.	1619.48	{1587.06, 1657.6}	57.1113	0.367718	newly	1211.	788.22	{754., 821.}	34.9116	0.186698	Reff	0.0154386	0.276709	{0.276137, 0.277354}	1692.32	ar	99540.	89664.3	{89613., 89712.}	9.92136
37	R0	{2.6202, 12.7552, 0.1}	{2.9884, 12.8326, 0.}	{{2.9884, 12.8326, 0.}, {2.9884, 12.8326, 0.}}	{14.0522, 0.606786, 100.}	inf	873.	1319.08	{1283.32, 1349.3}	51.0978	0.179179	newly	383.	581.93	{553., 607.}	51.9399	0.181348	Reff	0.016088	0.189237	{0.18874, 0.189867}	1076.26	ar	99426.	93969.4	{93940., 94001.}	5.48809
38	R0	{1.47672, 9.81651, 1.}	{2.52368, 12.5037, 0.}	{{2.52368, 12.5037, 0.}, {2.52368, 12.5037, 0.}}	{70.8975, 27.3743, 100.}	inf	569.	1373.55	{1336., 1409.86}	141.398	0.382637	newly	323.	631.87	{603., 659.}	95.6254	0.291169	Reff	0.0934468	0.275958	{0.275394, 0.276474}	195.31	ar	93815.	89570.3	{89519., 89621.}	4.52452
39	R0	{1.90004, 12.3313, 1.}	{2.64721, 11.9965, 0.}	{{2.64721, 11.9965, 0.}, {2.64721, 11.9965, 0.}}	{39.3241, 2.71487, 100.}	inf	1059.	1352.36	{1315.19, 1389.67}	27.7014	0.106114	newly	464.	639.11	{611., 666.}	37.7392	0.138799	Reff	0.0517191	0.249437	{0.248785, 0.250029}	382.292	ar	97413.	91013.4	{90970., 91058.}	6.5696
40	R0	{2.64218, 18.5237, 2.}	{2.99939, 13.7076, 1.}	{{2.99939, 13.7076, 1.}, {2.99939, 13.7076, 1.}}	{13.5196, 25.9995, 50.}	inf	2778.	1490.05	{1445.64, 1523.23}	46.3624	0.270617	newly	874.	619.33	{595., 649.}	29.1384	0.149905	Reff	0.0162494	0.18864	{0.188146, 0.189128}	1060.9	ar	99474.	94042.2	{94007., 94075.}	5.46057
41	R0	{1.6945, 7.17996, 0.1}	{2.17292, 6.16453, 0.}	{{2.17292, 6.16453, 0.}, {2.17292, 6.16453, 0.}}	{28.2334, 14.1425, 100.}	inf	103.	107.437	{93.7674, 121.189}	8.95118	0.0375866	newly	74.	86.02	{73., 98.}	17.7838	0.0695258	Reff	0.0626626	0.352865	{0.352574, 0.353122}	463.119	ar	96332.	83819.4	{83795., 83844.}	12.989
42	R0	{1.84559, 18.0074, 1.}	{2.81891, 18.2812, 0.}	{{2.81891, 18.2812, 0.}, {2.81891, 18.2812, 0.}}	{52.7374, 1.52076, 100.}	inf	3973.	4463.01	{4419.06, 4522.17}	12.3334	0.0504907	newly	1374.	1482.7	{1447., 1523.}	7.91121	0.032968	Reff	0.0586713	0.243345	{0.242463, 0.244051}	314.759	ar	97358.	92656.2	{92588., 92723.}	4.82936
43	R0	{2.24965, 17.0361, 2.}	{2.86001, 13.5542, 0.}	{{2.86001, 13.5542, 0.}, {2.86001, 13.5542, 0.}}	{27.1312, 20.4382, 100.}	inf	3123.	1997.96	{1955.89, 2034.45}	36.0244	0.194037	newly	1018.	851.64	{816., 891.}	16.3418	0.0777139	Reff	0.0319	0.215018	{0.214368, 0.215648}	574.038	ar	98770.	93005.5	{92964., 93055.}	5.83626
44	R0	{2.54324, 15.7967, 0.1}	{2.64721, 11.9965, 0.}	{{2.64721, 11.9965, 0.}, {2.64721, 11.9965, 0.}}	{4.0882, 24.0568, 100.}	inf	1624.	1355.96	{1321.32, 1393.26}	16.5047	0.0784308	newly	626.	639.49	{612., 665.}	3.29553	0.0140344	Reff	0.0177772	0.249476	{0.24892, 0.250068}	1303.34	ar	99354.	91010.6	{90959., 91060.}	8.39766
45	R0	{2.54943, 14.8032, 0.1}	{2.80387, 10.6536, 0.}	{{2.80387, 10.6536, 0.}, {2.80387, 10.6536, 0.}}	{9.98014, 28.0317, 100.}	inf	1364.	656.639	{630.809, 688.655}	51.8593	0.31772	newly	536.	336.31	{315., 360.}	37.2556	0.202972	Reff	0.0170047	0.214348	{0.213861, 0.214785}	1160.52	ar	99380.	92529.5	{92499., 92554.}	6.8932
46	R0	{2.37755, 12.6461, 2.}	{2.91946, 11.8546, 2.}	{{2.91946, 11.8546, 2.}, {2.91946, 11.8546, 2.}}	{22.7926, 6.25869, 0.}	inf	892.	1247.08	{1215.76, 1287.43}	39.8074	0.145424	newly	377.	586.5	{556., 616.}	55.5703	0.191583	Reff	0.0224678	0.199481	{0.198829, 0.200034}	787.851	ar	99096.	93468.8	{93426., 93513.}	5.67852
47	R0	{1.69279, 11.6636, 1.}	{2.52588, 12.2207, 0.}	{{2.52588, 12.2207, 0.}, {2.52588, 12.2207, 0.}}	{49.2143, 4.77662, 100.}	inf	1174.	1658.06	{1613.5, 1701.21}	41.2315	0.149844	newly	550.	773.24	{737., 805.}	40.5891	0.147714	Reff	0.0676608	0.278185	{0.277528, 0.278792}	311.146	ar	96214.	89600.2	{89549., 89650.}	6.87403
48	R0	{1.82402, 10.0038, 1.}	{2.74056, 10.6768, 0.}	{{2.74056, 10.6768, 0.}, {2.74056, 10.6768, 0.}}	{50.2483, 6.727, 100.}	inf	493.	831.264	{807.648, 853.055}	68.6135	0.226784	newly	248.	426.15	{405., 448.}	71.8347	0.234755	Reff	0.054447	0.227102	{0.226628, 0.227594}	317.106	ar	97076.	91950.8	{91916., 91986.}	5.27962
49	R0	{1.6424, 11.1667, 2.}	{2.71225, 12.7047, 1.}	{{2.71225, 12.7047, 1.}, {2.71225, 12.7047, 1.}}	{65.1393, 13.7732, 50.}	inf	672.	1189.48	{1153.24, 1224.07}	77.0061	0.247878	newly	321.	536.49	{506., 566.}	67.1308	0.222663	Reff	0.0752383	0.2353	{0.234685, 0.235759}	212.74	ar	95546.	91682.7	{91639., 91718.}	4.04337
50	R0	{2.05638, 14.3048, 1.}	{2.9884, 12.8326, 0.}	{{2.9884, 12.8326, 0.}, {2.9884, 12.8326, 0.}}	{45.3232, 10.2917, 100.}	inf	1589.	1317.01	{1284.68, 1348.83}	17.1171	0.0816034	newly	636.	580.99	{555., 602.}	8.64937	0.0395151	Reff	0.0397498	0.189114	{0.188599, 0.189691}	375.76	ar	98194.	93972.2	{93937., 94007.}	4.29943
